# *Bacillus* spp. as Bioagents: Uses and Application for Sustainable Agriculture

**DOI:** 10.3390/biology11121763

**Published:** 2022-12-05

**Authors:** Aimen Razzaq Khan, Adeena Mustafa, Sajjad Hyder, Mohammad Valipour, Zarrin Fatima Rizvi, Amjad Shahzad Gondal, Zubaida Yousuf, Rashid Iqbal, Umar Daraz

**Affiliations:** 1 Department of Botany, Government College Women University Sialkot, Sialkot 51310, Pakistan; 2 Department of Engineering and Engineering Technology, Metropolitan State University of Denver, Denver, CO 80217, USA; 3 Department of Plant Pathology, Bahauddin Zakariya University Multan, Multan 60000, Pakistan; 4 Department of Botany, Lahore College for Women University, Lahore 54000, Pakistan; 5 Department of Agronomy, Faculty of Agriculture and Environment, The Islamia University of Bahawalpur, Bahawalpur 63100, Pakistan; 6 State Key Laboratory of Grassland Agroecosystem, Center for Grassland Microbiome, College of Pastoral Agriculture Science and Technology, Lanzhou University, Lanzhou 730000, China

**Keywords:** *Bacillus* spp., biocontrol agent, sustainable agriculture, PGPR, biopesticide

## Abstract

**Simple Summary:**

To fulfill the food demand of the enormously growing population, different synthetic pesticides and fertilizers are used to grow crops. These synthetic products pose ill effects on humans and the environment. In recent times, the trend has shifted towards developing and utilizing bioproducts that are eco-friendly and sustainable to use in agriculture. They enhance productivity and restore equilibrium naturally in agroecological systems. In this regard, plant growth-promoting rhizobacteria (PGPR) facilitate crop production in multiple ways. This review deals with the limitations and challenges of conventional pesticides following the different microbes used as bioproducts along with how Bacillus is one of the promising PGPR used in sustainable agriculture. *Bacillus* spp. improves crop growth in both direct and indirect ways through nitrogen fixation, P and K solubilization, phytohormones production, quorum quenching, biofilm formation, and lytic enzymes production. Moreover, *Bacillus* spp. boost plant resistance towards the notorious phytopathogens. As *Bacillus* spp. is eco-friendly, promotes plant growth, confers resistance against diseases, improves soil fertility, non-toxic, naturally occurring microbe, and supports sustainable agriculture, there is a need to explore the potential of native *Bacillus* spp. and to use them in bioproduct development to support sustainable agriculture.

**Abstract:**

Food security will be a substantial issue in the near future due to the expeditiously growing global population. The current trend in the agriculture industry entails the extravagant use of synthesized pesticides and fertilizers, making sustainability a difficult challenge. Land degradation, lower production, and vulnerability to both abiotic and biotic stresses are problems caused by the usage of these pesticides and fertilizers. The major goal of sustainable agriculture is to ameliorate productivity and reduce pests and disease prevalence to such a degree that prevents large-scale damage to crops. Agriculture is a composite interrelation among plants, microbes, and soil. Plant microbes play a major role in growth promotion and improve soil fertility as well. *Bacillus* spp. produces an extensive range of bio-chemicals that assist in plant disease control, promote plant development, and make them suitable for agricultural uses. *Bacillus* spp. support plant growth by N fixation, P and K solubilization, and phytohormone synthesis, in addition to being the most propitious biocontrol agent. Moreover, *Bacilli* excrete extracellular metabolites, including antibiotics, lytic enzymes, and siderophores, and demonstrate antagonistic activity against phytopathogens. *Bacillus* spp. boosts plant resistance toward pathogens by inducing systemic resistance (ISR). The most effective microbial insecticide against insects and pests in agriculture is *Bacillus thuringiensis* (*Bt*). Additionally, the incorporation of toxin genes in genetically modified crops increases resistance to insects and pests. There is a constant increase in the identified *Bacillus* species as potential biocontrol agents. Moreover, they have been involved in the biosynthesis of metallic nanoparticles. The main objective of this review article is to display the uses and application of *Bacillus* specie as a promising biopesticide in sustainable agriculture. *Bacillus* spp. strains that are antagonistic and promote plant yield attributes could be valuable in developing novel formulations to lead the way toward sustainable agriculture.

## 1. Introduction

A range of plant diseases are caused by a variety of pathogenic microorganisms, such as fungi, bacteria, viruses, nematodes, and protozoa, which drastically lower agricultural production and cause significant yield losses [1]. Pathogenic diseases are responsible for between 20–40% of crop productivity losses [2]. Numerous methods have been employed to stop the spread of plant diseases, such as the use of pesticides, crop rotation, less susceptible crops, and other management techniques, but due to the resistance to pesticides and the persistence of soil-borne pathogens, their effectiveness is generally low [3]. Additionally, overusing chemically synthesized fertilizers has not only detrimental impacts on the biosphere but also affects the functioning of the ecosystem and diminishes the sustainability of agriculture [4]. Eco-friendly options for managing plant diseases and boosting crop yields are now being researched and advocated as part of an integrated crop management system—ICMS [5]. Biological control, which is a crucial component of ICMS, is described as the deployment of beneficial microorganisms to lessen the detrimental impacts of phytopathogens and encourage advantageous plant responses [6]. One of the most researched biocontrol agents, as biopesticides, is the *Bacillus* species, which inhibits phytopathogens by the mechanisms of competition and antagonism [7].

Various microorganisms, including *Hypericum gramineum*, *Pseudomonas fluorescence*, and *Streptomyces* species, have been identified as biocontrol agents [8]. *Bacillus* species have emerged as an important biological control agent because of their ability to produce antibiotics and tough and resistant endospores to control a range of phytopathogens [9].

Plant growth-promoting attributes have been reported in a variety of *Bacillus* spp., including *B. velezensis*, *B. subtilis*, *B. macerans*, *B. circulans*, *B. azotofixans*, *B. coagulans*, and others [10]. Phosphate solubilization, nitrogen fixation, production of siderophores, phytohormones, production of antimicrobial compounds, and systemically induced disease resistance are a few of the direct and indirect ways through which *Bacillus* spp. promote plant growth [11]. Antagonistically important species of the genus *Bacillus* are growing quickly. Abiotic stress resistance, rapid replication, and a broad spectrum of biocontrol capabilities are all characteristics of *Bacillus* spp. Volatile organic chemicals produced by *B. subtilis* are required for stimulating plant development and activating defense mechanisms in plants by boosting the induced systemic resistance (ISR) in plants [12]. Various crops, including tomato, potato, cucumber, maize, common [11] bean, soybean, sunflower, wheat, pepper, and many others, have shown positive impacts of *Bacillus* spp. on growth and crop yield [13].

## 2. Limitations and Challenges in the Use of Conventional Pesticides

Pesticides are noxious substances that are discharged premeditatedly into the environment to kill living beings, such as herbicides (kill weeds), insecticides (kill insects), fungicides (kill fungi), and rodenticides (kill rodents) [14]. Pesticide use has amazingly contributed in terms of both yield enhancement and the quality of the crop. The use of pesticides has become a widely adopted practice. According to an estimate, about 5.2 billion pounds of pesticides are used all around the world every year [15]. Although excessive or careless use of pesticides without following recommended practices and safety norms poses serious effects on living organisms (including human wellbeing) and the atmosphere [16]. Atreya, et al. [17] stated that “the benefit of pesticide use is yield increase.” However, a realistic approach must be broader and keep social and environmental impacts into account. Firstly, pesticide use may mitigate people’s wellbeing, reduce productivity, and increase medical expenses. Secondly, it is also involved in environmental or ecosystem degradation that increases costs indirectly.

Problems related to food scarcity, soil nutrient loss, and remnants of pesticides in the environment have put prodigious pressure on the ecosystem and wellness of humans [18]. Rajmohan, et al. [19] reported that “pesticides have a unique structure and their interaction with the environment characterizes the nature of pesticides.” In most cases, the end users, including farmers and consumers who have awareness and knowledge of the serious effects of pesticides, may be left using synthetic pesticides in their practice. The vigorous substances of pesticides cause pollution in the soil environment, affecting microbes living there. Wołejko, et al. [20] examined that the “imidacloprid application at heavy concentrations reduce bacterial population and gradually upset microbial balance in the soil.” Baćmaga, et al. [21] reported that chlorothalonil affects both the microbiological and biochemical properties of soil (loamy sand and sandy loam). It stimulated the growth of heterotrophic and actinobacteria that suppress the growth of fungi. Chlorothalonil acts as an inhibitor of acid phosphatase, catalase, and dehydrogenase activities. Hence, it is very important to minimize the usage of pesticides along with improved safety profiles to reduce deleterious effects on the well-being of humans and the environment. Moreover, there is a requisite to focus on what type of chemicals are the most auspicious for ecological and pest management.

## 3. Microbes as Sources of Biopesticides in Sustainable Agriculture (Biopesticides and Sustainable Agriculture)

**Figure 1:**
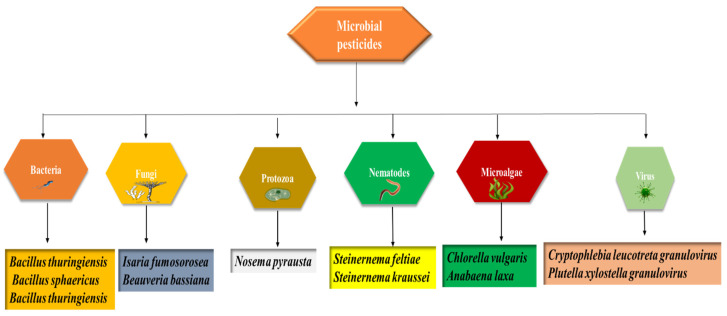
Sources of microbial-based biopesticides.

Biopesticides are obtained from microorganisms or natural sources [22]. They have been classified as follows; (a) botanical-biopesticides, (b) microbial-biopesticides, and (c) plant-incorporated protectants (PIPs). Sustainable agriculture aims to reduce pests and disease incidence to such an extent that it does not sternly damage crops without disturbing nature’s balance [23]. Although chemical pesticides expeditiously kill a range of agricultural pests, over-dependence on these pesticides has given rise to several problems involving safety risks, environmental pollution, secondary pests breakout, a decrease in biodiversity, and insecticide resistance [24]. Contrary to this, biopesticides are renewable and a significant alternative to conventional pesticides. The sources of microbial-based biopesticides are displayed in Figure 1.

They are helpful due to their less toxicity, eco-safety, specificity, no development of resistance to pests, and improved crop quality and production [25]. For example, Bt (*Bacillus thuringiensis*) is one of the biopesticides that is used globally as a tool for insect and pest control. It is also used in the management of phytopathogenic fungi via chitinolytic activity [26]. Kamarulzaman, et al. [27] carried out a comparative study to explore the effectiveness of biopesticides and conventional pesticides in paddy fields. In this study, a neem-based biopesticide (*Azadirachta indica*), its bioactive secondary metabolite (azadirachtin) bearing insecticidal properties, and conventional pesticides (niclosamideas) were tested. The productivity of rice (*Oryza sativa*) was increased by the application of biopesticides as compared to the conventional pesticide that offers an alternative for healthier rice cultivation. The primary aim of promoting biopesticides for sustainability is to establish a connection between socially admissible (health, culture, food security), economic growth (farming, marketing, income), and environmental stewardship (water, soil, climate, biodiversity). Along this configuration, the agriculture sector can attain feasibility, community welfare, and eco-safety [28].

## 4. Diversity of Species of the Genus *Bacillus* Existing in Agriculture Soil

**Figure 2:**
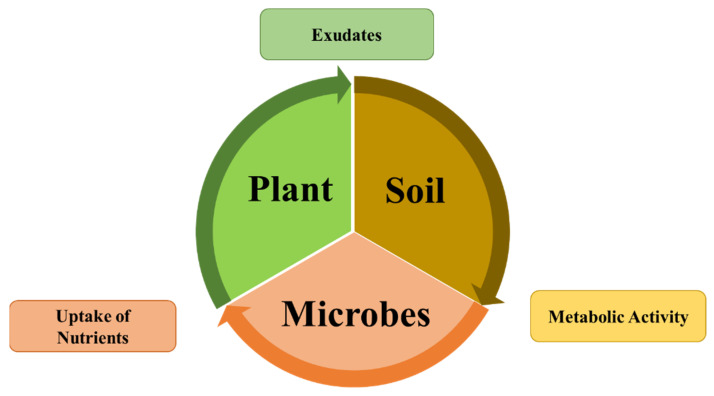
Interconnection between the plant, soil, and microbes. Plant roots secrete a prodigious diversity of organic nutrients and signals that attract microbes. Consequently, microbes breakdown the complex nutrients from complex to simple available forms to plants.

In the detention of soil, the enormous diversity of microbes, inclusive of bacteria species, archaea, and fungi, are precariously intricate with each other and involved in ecosystem functioning. According to an estimate, 1 g of soil may consist of 10^10^ to 10^11^ bacteria, 6000–50,000 bacterial species, and up to 200 m of myco-fungal hyphae, and most of them are propitious for plants and soils [29]. In recent days, intensive farming (by utilizing synthetic fertilizers and chemicals) has been the primary source of food for a growing population. These industrially composed chemical products cause eco-pollution and reduce the microbial population in the soil [30]. To secure biosafety, researchers are involved in the production of nutritious food under sustainable agriculture [31]. The interconnection between the plant, soil, and microbiomes is presented in Figure 2.

Various researches have reported the utilization of biofertilizers instead of hazardous chemicals. The microbes grown on synthetic culture are known as microbial inoculants or biofertilizers. These effective biofertilizers originated from beneficial bioagents that can improve soil fecundity as well as crop yield [31]. Extensive research has reported some probiotic bacteria such as *Bacillus*, *Pseudomonas*, *Enterobacter*, *Azotobacter*, *Serratia*, *Serratia*, *Arthrobacter*, *Erwinia*, *Microbacterium*, *Serratia*, *Azospirillum*, *Flavobacterium*, and *Caulobacter* [32]. The *Bacillus* species is one of the significant rhizobacteria species, such as *Bacillus subtilis*, *B. thuringiensis*, *B. cereus*, *B. pumilus*, etc., that promote plant growth and development and inhibit phytopathogens by the secretion of different exudates such as chitinase and beta-1,3 glucanase, etc. [33]. Some of the reported *Bacillus* species serving as biocontrol agents against various phytopathogens are presented in Table 1.

## 5. *Bacillus* spp. as PGPR (Plant Growth Promoting Rhizobacteria)

Bacteria that establish colonies in the rhizosphere (plant root zone) and boost plant growth are referred to as plant growth-promoting rhizobacteria—PGPR [48]. The bacterial strains of the genus *Bacillus* are among the most well-known PGPR [12]. *Bacillus* spp. is noted by rhizosphere residents and usually shows growth-promoting activities [49]. Certain *Bacillus* spp. enhances plant growth either by increasing the absorption of nutrients or by the activation of the host’s defensive mechanism against phytopathogens; in addition, other species can repress the population of pathogenic microbes [50]. These growth-promoting abilities of *Bacillus* can make it a competent PGPR and beneficent in sustainable agriculture [51]. A research study of Sansinenea [52] reported the inoculation of PGPR induced stress resistance as well as enhanced the yield in numerous crop species like tomato, lettuce, wheat, rice, soybean, groundnut, broad bean, maize, chickpea, etc. Various species, including *Bacillus azotofixans, B. subtilis, B. circulans, B. velezensis, B. coagulans, B. macerans,* etc., are reported as PGPR [53].

**Figure 3:**
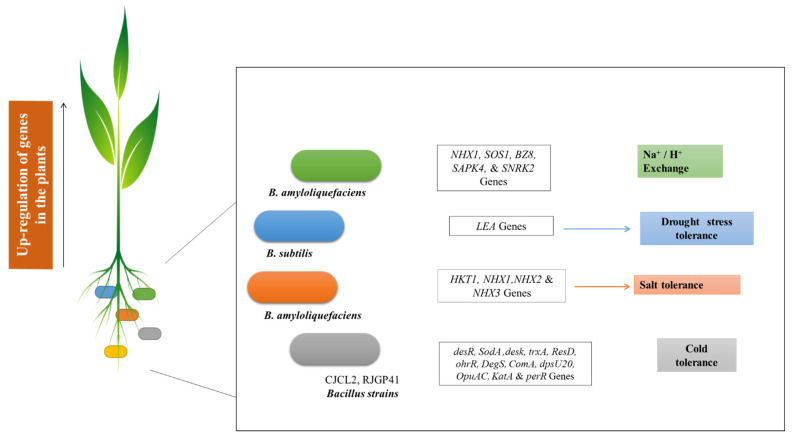
*Bacillus* spp. induced expression of genes in plants grown under various stress conditions.

Presently, a change in the world’s climate and deleterious environmental conditions are playing an important role in the reduction of crop development, growth, and yield [54]. The manifestation of new varieties of crops (against stress) by implying genetic engineering and molecular breeding is costly and labor-intensive. So recently, the usage of growth-promoting rhizobacteria has been gaining huge popularity as an alternative approach for amelioration of stress in different crops [55]. Accordingly, Nautiyal, et al. [56] observed PGP traits of *B. amyloliquefaciens* and its impact on *Oryza sativa* grown under salt-stress conditions. Salt-responsive genes, including *NHX1*, *SOS1*, *BZ8*, *SAPK4*, and *SNRK2*, have been reported in rice plants. The *NHX1* and *SOS1* were reported to be involved in Na^+^ and H^+^ exchange, respectively, and reduced cellular Na^+^ ion concentration. The *SAPK4* gene controls ion homeostasis, improved growth, and development of plants under salt stress. The *SNRK2* and *BZ8* function in the ABA gene regulation pathway through osmotic signaling. In *Brachypodium distachyon* the application of *B. subtilis* increases the expression of *LEA* genes responding to drought stress [57]. Chen, et al. [58] reported *B. amyloliquefaciens* inoculation is helpful in the expression of genes that help maize plants against salt stress. The *HKT1*, *NHX1*, *NHX2*, and *NHX3* genes are related to ion balance and assist maize plants in becoming salt tolerant. Another study by Zubair, et al. [59] highlighted two *Bacillus* strains, CJCL2 and RJGP41, for their potential role in mitigating cold stress and fostering plant growth in wheat plants. The reported genes for cold tolerance are *DegS*, *desR*, *SodA*, *trxA*, *dpsU20*, *ResD*, *ohrR*, *desk*, *ComA*, *OpuAC*, *KatA*, and *perR*. The pictorial representation of *Bacillus* spp. induced gene expression in plants under various stress conditions is presented in Figure 3.

## 6. Mechanisms of PGPR

**Figure 4:**
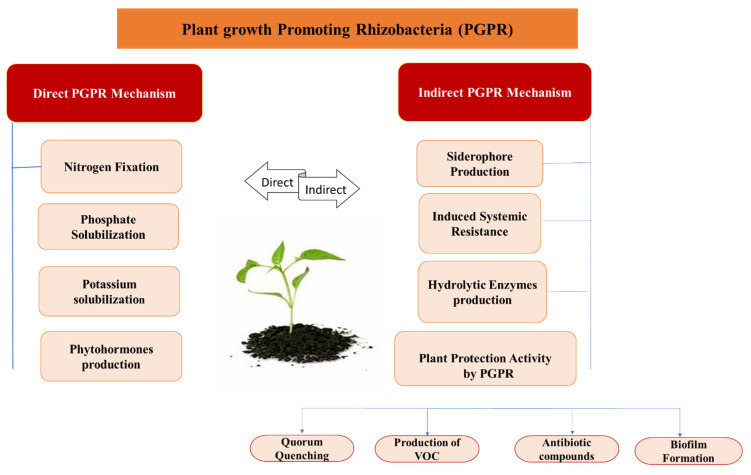
Direct and indirect mechanisms of plant growth promoting activities of PGPR.

Plant growth-promoting bacteria, through various direct and indirect mechanisms, suppress the phytopathogens and promote plant growth, as presented in Figure 4. These mechanisms are discussed in detail below:

### 6.1. Direct Mechanism of PGPR

Several PGPR promote plant growth in various ways. The direct PGPR mechanisms that affect plant growth are described below:

#### 6.1.1. Nitrogen Fixation

The capability of *Bacillus* spp. to produce different types of metabolites has a direct impact on plant development and agricultural yield by boosting the nutrients that are available to the plants. Most of the required nutrients are applied to plants by fertilization. This technique not only causes greater economic loss but also produces a harmful impact on the surroundings. Biofertilizers containing phosphorous solubilizing and nitrogen-fixing *Bacillus* spp. are an acceptable way to minimize dependency on chemical fertilizers while maintaining food safety [60]. Despite being primarily (more than 80%) inaccessible in its atmospheric form, nitrogen (N) is essential for the optimal development of plants [61]. Biological nitrogen fixation (BNF) is a phenomenon in which microorganisms absorb elemental nitrogen from the environment and convert it into a compound that is used by plants as nutrients [62]. Nitrogenase, an enzyme produced by nitrogen-fixing bacteria, catalyzes molecular dinitrogen (N_2_) to ammonia (NH_3_) and then is absorbed by the roots of plants. BNF produces approximately 200 million tons of nitrogen annually in the ecosystems of the earth. The microorganisms that are involved in nitrogen fixation may be free-living or symbiotic in nature. By engaging in a-symbiotic nitrogen fixation, several *Bacillus* spp. can reduce the dependence on nitrogen-based synthetic fertilizers while promoting plant growth along with crop productivity. In crops, 12–70% of total nitrogen uptake is possible due to BNF [61]. In a research study, Kuan, et al. [63] examined the efficacy of *B. pumilus* as an atmospheric nitrogen fixer, the rise in nitrogen contents, and dry biomass in the maize plant.

#### 6.1.2. Phosphate Solubilization

Phosphorus (P) is a primary macronutrient that is crucial for the development and growth of plants [64]. P-solubilization is defined as the mobility of bound inorganic P (P_i_) through the desorption of P and dissolution of P-containing minerals, such as apatite (the group of phosphate minerals) [65]. Alori, Glick and Babalola [64] reported the excessive utilization of synthetic P fertilizers to uplift agricultural production to fulfill ever-increasing global food demand has the potential to pollute surface and groundwater, eutrophication of waterways, and deplete soil fertility. A variety of soil microbes are capable of solubilizing organic P into P_i,_ which can then be used by plants. These microbes enhance the growth as well as production of a broad range of crops. Saeid, et al. [66] examined the phosphorus solubilization ability of three selected bacterial strains belonging to *Bacillus* spp. (*B. cereus, B. subtilis*). *Bacillus* produced solubilizing exudates including five organic acids namely acetic, gluconic, succinic, lactic, and propionic acids. The presence of phosphorus was established. However, a coalition of three *Bacillus* spp. produced a significant quantity of organic acid content.

#### 6.1.3. Potassium Solubilization

Potassium is the third fundamental plant macronutrient, following nitrogen and phosphorus [67]. This element takes part in several physiological and metabolic processes of the plants, including photosynthesis, stomata regulation, proper seed development, and promoting crop growth and yield [68]. In soils, K-containing minerals that discharge K through weathering are feldspar, muscovite, biotite, alkali, and illite [69]. However, a major dilemma is the unavailability of these minerals (for plants), but with the assistance of K solubilizing microorganisms inclusive of bacteria, actinomycetes, and molds, improved potassium dissolution can be achieved. The acid production by the bacteria is the mechanism by which K is released, such as *Bacillus mucilaginosus* and *Priestia megaterium* are the reported *Bacillus* spp. that assist in K dissolution [70]. Ali, et al. [71] observed the response of *Solanum tuberosum* plants to a biofertilizer containing *Bacillus cereus* investigated in a two-year field study. These potassium-solubilizing bacteria improve the growth parameters of potato plants more than untreated plants. Bacterial inoculation presents a notable increase in plant height and dry biomass. In addition to this, bio-fertilizers improve the total yield and the graded weight of potato tubers. An extensive range of bacteria, such as *B. circulans* and *B. edaphicus* has been analyzed to liberate potassium from minerals to a usable form available to the plants [33].

#### 6.1.4. Phytohormones Production

Chemical messengers that are mediated in biochemical and physiological processes of higher plants that are active at very low concentrations refer as phytohormones [72]. Charles Darwin was the first person who suggested that certain chemical compounds capable of stimulating growth in crops are latterly known as phytohormones. Microorganisms can stimulate growth and enhance the resistance of plants by synthesizing phytohormones [73]. Plant roots are heavily surrounded by microbes because of root exudates that are rich in nutrient components [74]. Classical bacterial-phytohormones are ethylene, cytokinins, auxins, abscisic acid, and gibberellins [75]. In a research study, Kang, et al. [76] reported that due to climate change, the crops also experience a change in temperature that is drastically damaging crop growth and development. The PGPR professes an appealing strategy to counter the heat stress that causes detrimental effects on the crops. Accordingly, *B. tequilensis* (SSB07) is an actively growing bacterial strain in the rhizosphere of Chinese Cabbage that improves the growth of cabbage seedlings by producing a variety of gibberellins such as GA_5_, GA_1,_ GA_19_, GA_53_, GA_8_, GA_24_, and GA_3_, as well as abscisic acid and IAA (indole3-acetic acid).

Auxins are a group of hormones that incite tissue differentiation, cell elongation, and cell division in plants [61]. IAA is a predominant auxin in plants that was chemically identified in the 1930s [77,78]. There is also much evidence documented that various soil microorganisms laboriously synthesize auxins in pure culture and in soil [78]. Ahmed and Hasnain reported in 2010 that two strains (P4 and S6) of the *Bacillus* spp. ameliorated the auxin content in inoculated plants observed by up to 433% as compared to 71.4% in the non-inoculated plants of *S. tuberosum*. Comparability of inoculated and non-inoculated plants disseminated, approximately 35% to 40% escalation in the shoot lengths and 40% and 50% increase in the root lengths by P4 and S6 strains respectively. Briefly, auxin-producing *Bacillus* spp. influence the stimulative effects on the development of plants.

Gibberellins (GAs) are a class of hormones that endeavor profound effects on the growth and development of plants [79]. GA act as a key for following processes in plants, including root and shoot elongation, seed germination, flowering, and fruiting pattern [80]. Further studies revealed GA as a crucial hormone in plants for many developmental processes, such as a transition from vegetative to reproductive growth; trichome development; flower, seed, and fruit development [81,82,83]. The findings of Khan, et al. [84] suggested that *B. cereus* could be used as a growth promoter and thermo-tolerant in soybean plants. In another study, the synthesis of four gases by *B. licheniformis* and *B. pumilus* has been described [85].

Both *B. subtilis* and *B. licheniformis* exhibit the synthesis of bacterial cytokinins [86]. Cytokinins are plant hormones that play a key role in physiological and growth-related processes [87]. Stomatal opening and an increase in shoot growth are produced by cytokinins [88]. Cytokinins take part in a variety of plant growth processes, inclusive of photosynthesis, chloroplast differentiation, cell division, regulation of leaf senescence, and nutrient metabolism [89]. Cytokinins presumably arrived from the root zone; however, they accumulate predominantly in the shoots but not in inoculated plants’ roots [90].

Ethylene is a simple hydrocarbon with gaseous nature. Additionally, it regulates stress responses, seedling growth, seed germination, leaf and petal abscission, organ senescence, and pathogen responses [91]. As plants are exposed to various environmental stresses, they speed up their ethylene production rate [92]. Interestingly, ethylene is different and unique due to its gaseous nature; likewise, it moves within the plant by diffusion and considers to be synthesized at or near its site of action [93]. Misra and Chauhan [94] stated that, generally, “salt stress actively corresponds with better ethylene production.” Salt-tolerant *Bacillus* strains synthesize ACC (an immediate precursor of ethylene) deaminase activity that raises the ethylene level in plants grown under salt stress and results in the growth promotion in *Zea mays.*

Stomatal closure, fruit ripening, and seed germination are the vital functions of Abscisic acid (ABA). Moreover, it is also engaged in bud dormancy and protective responses against abiotic stresses (salt and drought stress and heavy metal toxicity) [95]. According to Shahzad, et al. [96], salinity hinders crop yield and plant growth. The bacterial strain *B. amyloliquefaciens* has been observed to produce ABA and has the potential to increase resistance against salt stress in rice plants (*Oryza sativa*).

### 6.2. Indirect Mechanism of PGPR

The indirect mechanism of PGPR involved the following attributes, as explained below.

#### 6.2.1. Siderophore Production by *Bacillus* spp.

Siderophores are low molecular weight, metal-chelating compounds that are produced under iron-limited conditions by some microbes and plants [97]. Iron (Fe) acts as a key element in various kinds of biological processes, e.g., metabolism of oxygen, synthesis of DNA and RNA, transfer of electrons, and enzymatic processes. Siderophores have the capability to lessen the accessibility of Fe for pathogens [98]. By functioning as biocontrol agents, microbes that create siderophores can restrict the spread of diseases and promote the growth of the plant [11]. Out of various *Bacillus* spp., *B. licheniformis, B. anthracis, B. velezensis, B. thuringiensis, B. cereus, B. halodenitrificans, B. atrophaeus, B. mojavensis, B. pumilus,* and *B. subtilis* are the well-known for siderophore production [99]. Siderophore is induced by numerous species of *Bacillus,* and these species actively participate in the reduction of different plant diseases. For instance, *B. subtilis* produced a siderophore, which was involved in the reduction of *Fusarium* wilt and increased the pepper yield [100]. Moreover, siderophores, as bioremediation agents, have the potential to bind different kinds of metals present in the environment [101]. Chelators and phyto-siderophores are two examples of the main metabolites produced by *Bt* strains that are involved in plant development [26].

Bt produces bacillibactin, a siderophore of the catecholate type that binds iron with an extremely great affinity. Siderophores might help the plant by delivering iron, or they might inhibit the growth of phytopathogenic fungi by competing for iron with Bt strains [102].

#### 6.2.2. Induced Systemic Resistance—ISR

Non-pathogenic rhizobacteria have the capacity to lessen diseases in plants by mediating a plant defense process called “Induced Systemic Resistance” (ISR) [98]. It takes a combination of biotic and abiotic stimuli for plants to start developing the ISR (mechanism of resistance). Non-pathogenic rhizobacteria participate in mediating ISR and usually rely on the ethylene (ET) or jasmonate (JA) signaling pathways [103], while Systemic Acquired Resistance (SAR) is promoted through the help of salicylic acid (SA). SAR is responsible for the stimulation of a particular group of defense-related genes, while ISR is not involved in the triggering of any certain kinds of defense-related genes [104]. PGPR induces ISR in plants by releasing various metabolites, e.g., antibiotics, siderophores, volatile organic compounds (VOCs), etc. Through the release of these compounds, PGPR can trigger the mechanism of ISR in plants. *Bacillus* spp. can initiate ISR by the production of antioxidant defense enzymes. Different defense-related enzymes, e.g., polyphenol oxidase (PPO), superoxide dismutase (SOD), peroxidase (POX), and phenylalanine ammonia-lyase (PAL), are induced by *B. subtilis*. In tomato seedlings, the prolonged formation of antioxidant defense enzymes induces the mechanism of ISR against early and late blight diseases [105]. Some strains of *Bacillus* spp. reduced the chili anthracnose disease by the formation of phenolic compounds and induction of antioxidant defense enzymes [106]. In a related research study, Chen, et al. [107] examined that *B. subtilis* has the capability to inhibit disease incidence, increase the growth of seedlings, and enhance the defense-related enzyme activities in the cucumber plant. Similarly, Jain, et al. [108] observed that *Bacillus* spp. enhanced plant growth promotion activities and inhibited disease in soybean caused by *Fusarium oxysporum* and *Rhizoctonia solani* by the induction of defense-related enzymes (POX, PAL, PPO).

#### 6.2.3. Production of Lytic Enzymes

Lytic enzyme production is an intrinsic characteristic of biocontrol agents in the prevention of disease-causing microbes [109]. The activity of lytic enzymes disrupts the cell walls of targeted pathogens by changing the structural stability and integrity [110]. Chitin is a major constituent of the cell walls of fungi, among other composition molecules [111]. Some bacterial strains (PGPR) can degrade fungal cell walls by producing hydrolytic enzymes, including chitinases, dehydrogenases, exo- and endo-polygalacturonases, lipases, phosphatases, proteases, β-glucanases, hydrolases, pectinolyases, and cellulases. Another study reported that the synthesis of lytic enzymes might also be helpful for bacteria to penetrate plant tissues and grow as endophytes [112]. Moreover, some of the *Bacillus* strains were reported as the producer of proteases that are helpful in the degradation of the cuticle of the nematodes. Santoyo, Urtis-Flores, Loeza-Lara, Orozco-Mosqueda and Glick [111] studied *Bacillus thuringiensis*, producing chitinases against *B. cinerea*, the causal agent of gray mold. Karthika, et al. [113] elucidated *B. cereus* and *Burkholderia cepacia* produce cellulase, β-1,3-glucanase, amylase, protease, lipase, and xylanase as a factor in the rupturing of certain soil-borne pathogenic microorganisms’ cell walls.

## 7. Plant Protection Activity Stimulated by *Bacillus* spp.

Strains of the *Bacillus* spp. are used as biological control agents (BCAs) to protect plants from pathogenic diseases. Chemical pesticides are being replaced by BCAs, which is a viable option. As a result, various researchers are focusing on exploring their interactions with pests, plants, and pathogenic and beneficial microbes, as well as their environmental impact and human implications. Important characteristics, including efficacy, formulation, stability, and viability, were all thoroughly investigated in many studies. There are several mechanisms by which PGPR protects plants against diseases. These mechanisms are discussed below:

### 7.1. Quorum Quenching

Communication inside the bacterial population is feasible with the help of quorum sensing molecules, N-acyl homoserine lactone (AHL). Such indicating molecules are the main reason for boosting the infectious diseases in the pathogenic microbes. Those microorganisms which release AHL lactonase enzyme behave as a biocontrol agent. AHL lactonase is an enzyme that hinders bacterial communication systems by breaking down the quorum-sensing signaling molecule. Quorum quenching was noticed in different *Bacillus* spp., including *B. cereus*, *B. thuringiensis*, and *B. licheniformis* [114].

### 7.2. Production of Volatile Organic Compounds (VOCs)

Lower molecular weight lipophilic compounds with high vapor pressure and low boiling point are released by microbial metabolic processes. VOCs function as signal molecules both over short and long distances in the rhizosphere [115]. Additionally, 2,3-butanediol is a volatile organic compound produced by *B. subtilis* engaged in the mechanisms of plant defense. Phytopathogens were challenged by using the root exudates from peppers inoculated with *B. subtilis*. For example, volatile organic compounds were responsible for the growth inhibition of *Trichoderma* spp. and *Ralstonia solanacearum*. So, this study revealed that volatile organic compounds caused the excretion of root exudates and ultimately worked as an inducer of plant defense against soil-borne bacterial and fungal diseases [116].

### 7.3. Antibiotic Compounds

Antibiotic production by beneficial microorganisms [86] is the most effective biological control method for controlling plant diseases. Such chemicals are secreted by *Bacillus* spp. during sporulation and the stationary development stages [86]. Bacitracin, Kanosamine, fengycin or plipastatin, surfactins, zwittermicin A, kurstakin, gramicidin, and iturins are important antibiotic compounds produced by *Bacillus* spp. Bacitracin is another kind of antibiotic compound that has strong bactericidal activity. Different *Bacillus* spp., including *B. subtilis* and *B. licheniformis*, have been found to synthesize bacitracin [114]. Kanosamine is an antibiotic compound that has a high inhibitory effect on fungal pathogens of oomycetes and a moderate effect on Ascomycetes, Basidiomycetes, Deuteromycetes, and some bacteria. Production of Kanosamine was reported in several species of *Bacillus*, including *B. pumilus* and *B. subtilis* [117] or *B. cereus*. Fengycin A and B are also known as plipastatinare lipopeptide antibiotics. These antibiotic substances played a very active role in controlling mosquito larvae [118] and phytopathogens by breaking down their cellular structure and cell membrane permeability [119]. These compounds also help in the stimulation of the induced systemic resistance (ISR) pathway in plants [120]. They are used as biosurfactants and display the ability to breakdown the polycyclic aromatic hydrocarbons (PAHs) [121] in several bacteria [122]. These antibiotic compounds are synthesized by various *Bacillus* spp. including *B. amyloliquefaciens*, *B. subtilis*, and *B. licheniformis* [107].

### 7.4. Biofilm Formation by Bacillus spp.

In the past, induction of systemic resistance and synthesis of antimicrobial compounds were two reported methods that biocontrol agents utilize to combat phytopathogens. However, current research in the field of biocontrol has focused on biofilm formation and root colonization as defense mechanisms against biocontrol activity. Several *Bacillus* spp. including *B. velezensis*, *B. atrophaeus*, and *B. subtilis* have been reported to colonize roots and create biofilms as a biocontrol strategy. In many *Bacillus* species, plant root exudates and various lipopeptides, including bacillomycin and surfactin, play a vital role in the formation of *biofilm* [123]. For instance Fan, Wang, Song, Ding, Wu, Wu, Gao and Borriss [10] reported that *B. velezensis* strain FZB42 induces biofilms in the roots of corn, mouse-ear cress, and duckweed. It was further examined that surfactin (lipopeptide) played a key role in root colonization and stimulation of biofilm synthesis in *Bacillus* spp. like *B. atrophaeus* and *B. subtilis*.

## 8. Multifaceted Role of *Bacillus thuringiensis* as a Biocontrol Agent

*Bacillus thuringiensis* (*Bt*) is an entomopathogenic bacteria that create parasporal crystal proteins (δ-endotoxins). These δ-endotoxins are poisonous to Lepidoptera, Coleoptera, and Diptera, among other insect pests [124]. Throughout the previous century, *Bt* has been regarded as the most effective bioinsecticide [125]. Because *Bt* is a rapid-acting and host-specific bioinsecticide, it has few side effects on non-target organisms. Furthermore, its production and use are simple and inexpensive [126]. To generate transgenic crops that are resistant to pests, plant genetic engineering has successfully used *Bt* as a source of Cry genes [127].

The production of bacteriocins is the main antimicrobial activity of the Bt strain [125]. To strengthen the defense against different microorganisms, prokaryotes frequently produce a variety of antimicrobial peptides. Bacteriocins are tiny, thermotolerant antimicrobial peptides produced by ribosome synthesis in the stationary phase, with molecular weights ranging from 3 to 12 kDa. In a study, de la Fuente-Salcido, et al. [128] reported various types of bacteriocin produced by Bt strains. From the Bt subspecies, more than 18 different forms of bacteriocin have been identified and purified, including thuringiensis, morrisoni, tochigiensis, kurstaki, tolworthi, kenyae, and entomocidus. Bacteriostatic or bactericidal actions of Bt bacteriocins might be broad or specific.

Various Bt strains can compete with plant pathogenic bacteria through the production of various bacteriocins and AHL-degrading enzymes. AHL-degrading enzyme (AiiA), released by some Bt strains, can reduce the virulence of pathogenic bacteria like *Erwinia carotovora*, which causes soft rot in the roots of *Capsicum annuum* [129]. Furthermore, the inclusion of vegetative cells of Bt in combination with other bacterial (*Streptomyces avermitilis* and *Citrobacter farmeri*) and fungal (*T. viride*, *T. parareesei,* and *Paecilomyces variotii*) antagonists significantly increased their effectiveness to suppress *Ralstonia solanacearum* in *Capsicum chinense* [130] and *S. lycopersicum* [131]. Another study highlighted that the mixture of Bt, *T. viride*, and *T. parareesei*, demonstrated the strongest antagonistic effect (91.47%) against *R. solanacearum* [130].

*Bacillus thuringiensis* (Bt) produces crystal proteins (Cry), also called δ-endotoxins. *Bacillus* produces the most prominent group of insecticidal proteins, which are known as cry toxins. According to the nomenclature committee of Bt toxin, 78 distinct Cry toxins have been identified to date, with Cry1 being the most common [132]. A wide range of *B. thuringiensis* subspecies produces a variety of Cry toxins. *B. thuringiensis* var kurstaki produces 31 distinct forms of Cry proteins, the most common of which are Cry1Aa and Cry1Ac. *B. thuringiensis* israelensis is the main producer of Cry4, Cry10, and Cry11 toxins. Cry1 toxins are mostly active against Dipterans, Lepidopterans, and Coleopterans, whereas Cry2 toxins are mostly poisonous to Dipterans, Lepidopterans, and Hemipterans. Although there are many Cry proteins, however, only Cry1 has been economically utilized. There are a variety of commercial Bt bioinsecticides present in the market (e.g., Dipel, Thuricide, Biobit, Gnatrol, VectoBac, and so on).

The use of nanotechnology has enabled the study of Bt-based biopesticides to go to a new stage. The greater efficacy of nanotized Bt products has been noticed [53]. In a research study, Murthy, et al. [133] synthesized Bt powders with 32~1100 nm-sized particles, which had a greater mortality rate. Vineela, et al. [134] created Bt particles ranging in size from 105 to 210 nanometers that had insecticidal activity against *Spodoptera litura* as compared to the synthetic insecticide Profenophos. Cry toxins can be placed onto nanomaterials instead of being nanotized from Bt powders. In a study, Cry1Ac protein was filled with magnesium hydroxide nanoparticles, which enhanced the mortality rate of *Helicoverpa armigera* and boosted the adherence to cotton leaf surfaces [135]. Bt, the bacterial system that has received the greatest attention in the sector of plant biotechnology, is used to produce crop plants that are resistant to insects as well as biopesticides. Several crops, including *Zea mays*, *Gossypium herbaceum*, and *S. tuberosum* have been genetically modified to get improved yields [136].

## 9. Biosynthesis of Metallic Nanoparticles by *Bacillus* spp.

According to Xu, et al. [137], bioinspired synthesized nanoparticles can be eco-friendly compared with chemical synthesis and attracting much attention from researchers in recent years. Microbes, including bacteria, can synthesize nanoparticles extracellularly or intracellularly during incubation time after cultivation. These creatures reduce the toxicity of metal ions by consuming them as nutrition and reducing the metal ions to metal nanoparticles by various metal ion reductases. These reductases assist in awarding the bio nanoparticles more substantial functionally and provide stability. In a research study, Jeevanandam, et al. [138] prepared silver nanoparticles from *B. amyloliquefaciens*, *B. cereus*, *B. indicus*, and *B. cecembensis,* while *B. subtilis* and *P*. *megaterium* were used in the fabrication of gold nanoparticles. Ahmed, et al. [139] reported *B. cereus* strain is supportive in the concoction of ZnO_2_ nanoparticles that exhibit activity against *Burkholderia glumae* and *B. gladioli* which caused panicle blight in *Oryza sativa*.

## 10. Effect of *Bacillus* spp. on Uptake of Nutrients and Crop Yield

Meena, et al. [140] studied that the *Bacillus* spp. enhances the yield of various crops, e.g., wheat, maize, sugar beet, and spinach. Verma, et al. [141] observed the potency of *Bacillus* strains with the combination of other rhizobacteria on bean growth and yield, which had a substantial impact on nodule development in pulse crops. Similarly, ČOlo, et al. [142] reported that *B. subtilis* increased the growth parameters as well as the yield of the onion crop by producing IAA. Mukhtar, Shahid Mukhtar, et al. [143] examined the ability of *B. safensis* and *P. megaterium* to enhance plant growth parameters, e.g., dry weight of root, shoot, and weight of seed in wheat crop. Vinci, et al. [144] observed that the co-inoculation of *Bacillus velezensis* FZB42 and compost significantly improves growth parameters as well as increases the uptake of nutrients in the maize plant. Photosynthetic activity is also boosted in the leaves of maize plants due to the enhancement of the synthesis of metabolites, gamma-aminobutyric acid (GABA), alanine, glucose, and fructose. The effect of *Bacillus* spp. on crop growth, crop yield, and uptake of nutrients with various crop species is presented in Table 2.

## 11. Conclusions

Pesticides have been proven to be a promising agent to fulfill the food demand of the growing population. However, these hazardous pesticides have caused human health problems, development of pest resistance, narrowing of biodiversity, and environmental challenges, raising concerns about the pesticides’ safety. Thus, the need to reduce reliance on these synthetic pesticides is pertinent. The application of PGPR is an auspicious solution for eco-friendly agriculture. *Bacillus* spp. have been elucidated as growth promoters in sustainable agriculture through both direct and indirect mechanisms. The N_2_-fixation, P and K Solubilization, phytohormones production by *Bacillus* strains, moreover synthesis of antibiotics, production of lytic enzymes, and ISR are direct and indirect mechanisms, respectively, and all these action mechanisms of *Bacillus* are supportive in the growth promotion of plants, pest resistance, and circumventing of disease. Some of the *Bacillus* spp. have been documented as promising biocontrol agents. Food production and its accessibility always are an overwhelming priority to feed the world’s population. So, the best route is to be cautious about chemical-based pesticides. Biopesticides have long been attracting global attraction due to their safer strategy than conventional pesticides. Considering the importance of sustainable agriculture [173,174,175,176], *Bacillus* spp.-based bioproducts could be a promising addition to sustainable agriculture as there is a limited product range available. There is a dire need to explore the potential of *Bacillus* spp. in combination with other compatible microbial agents to increase PGP activity and quality food production.

## Figures and Tables

**Table 1:** *Bacillus* species were reported as biocontrol agents against various phytopathogens.

*Bacillus* Species	Plant Species	Pathogens	References
*Bacillus subtilis*	Wheat	*Rhizoctonia cerealis*	[34]
*Bacillus velezensis*	Pear fruits	*Apergillus westerdijkiae*	[35]
*Bacillus amyloliquefaciens*	Rice grains	*Aspergillus westerdijkiae*	[36]
*Bacillus cereus*	Potato	*Fusarium oxysporum*	[37]
*Bacillus subtilis*	Tomato	*Pythium ultimum*	[38]
*Bacillus spp.*	*-*	*Rhizoctonia solani*	[39]
*Bacillus velezensis*	*-*	*Fusarium oxysporum, F. graminearum, Botrytis cinerea, Alternaria alternata, Fulvia fulva, and Ustilaginoidea virens.*	[40]
*Bacillus amyloliquefaciens*	Tomato	*Fusarium oxysporum*	[41]
*Bacillus amyloliquefaciens* and *Bacillus subtilis*	Tomato	*Botrytis cinerea*	[42]
*Bacillus amyloliquefaciens*	*Mustard*	*Sclerotinia sclerotiorum*	[43]
*B. vallismortis, B. amyloliquefaciens and B. thuringiensis*	*Eggplant*	*Ralstonia solanacearum*	[44]
*Bacillus* spp.	*Sweet pepper*	*Phytophthora capsici*	[45]
*Bacillus velezensis*	Maize crop	*Fusarium graminearum* and F. *culmorum*	[46]
*Bacillus velezensis*	Pepper	*Botrytis cinerea*	[47]

**Table 2:** Multifaceted impacts of *Bacillus* sp. on various crops grown under various stress conditions.

*Bacillus* spp.	Plant Species	Impact	References
*Bacillus licheniformis*	*Zea mays*	Drought tolerance	[145]
*Bacillus pumilus*	*Triticum aestivum*	PGPR under salinity stress	[146]
*Bacillus cereus*	*Solanum nigrum*	IAA producer	[147]
*Bacillus velezensis*	*Solanum lycopersicum*	Biofilm formation	[148]
*B. subtilis*	*Phaseolus vulgaris*	Bio fertilizer	[149]
*B. pumilus*	*Triticum aestivum*	Biofilm formation	[150]
*Bacillus pumilus*	*Fagopyrum esculentum*	Antifungal impact	[151]
*Bacillus amyloliquefaciens*	*Solanum tuberosum*	Disease management	[152]
*Lysinibacillus fusiformis*	*Cicer arietinum*	Anti-fungal activity	[153]
*Bacillus mycoides*	*Lolium perenne*	PGPR	[154]
*Priestia megaterium*	*Phaseolus vulgaris* L.	Mitigate salinity stress	[155]
*Paenibacillus polymyxa* and *Bacillus circulans*	*Zea mays*	Copper stress tolerance	[156]
*Bacillus thuringiensis*	*Gossypium herbaceum*	Genetically modified crop (insecticide)	[157]
*Bacillus subtilis*	*Lycopersicon esculentum, Zea mays*	Biofilm formation ameliorates water stress	[158,159]
*Bacillus methylotrophicus*	*Lactuca sativa*	GAs production	[160]
*Bacillus pumilus*	*Zea mays*	N_2_ –fixation	[63]
*Bacillus aryabhattai*	*Glycine max*	Phytohormones (ABA, IAA, CKs, GAs) production	[161]
*Bacillus subtilis*	*Arabidopsis thaliana* and *Brassia campestris*	Drought and salt stresses	[162]
*B. subtilis*	*Manihot esculenta*	Acts as PGPR and promotes biomass	[163]
*B. amyloliquefaciens*	*Musa paradisiaca*	IAA	[164]
*Bacillus megaterium*	*Solanum melongena*	P-Solubilization	[165]
*Bacillus thuringiensis, P. megaterium* and *Bacillus subtilis*	*Cicer arietinum*	Drought tolerance	[166]
*Bacillus subtilis*	*Triticum aestivum* L.	Alleviate drought stress	[167]
*Bacillus cereus, Bacillus velezensis* and *Bacillus thuringiensis*	*Lycopersicon esculentum*	PGPR	[168]
*Bacillus sonorensis*	*Capsicum annuum* L.	P-solubilizer, siderophore, chitinase, IAA, hydrogen cyanide, and biofilm formation	[169].
*Bacillus firmus* and *Bacillus amyloliquefaciens*	*Zea mays* and *Glycine max*	PGPR	[170]
*B. thuringiensis*	*Lavandula dentate*	Drought tolerance	[171]
*Bacillus licheniformis, Bacillus subtilis, Bacillus amyloliquefaciens, Bacillus mycoides, Bacillus methylotropicus*	*Cucumis sativus* L.	Reduce salinity stress	[172]

## Data Availability

Not applicable.
